# Association between the geriatric nutritional risk index and dysphagia in older critically ill patients: A retrospective analysis of the MIMIC-IV database

**DOI:** 10.1097/MD.0000000000051456

**Published:** 2026-11-06

**Authors:** Chong Wang, Lili Lv, Rongrong Ma

**Affiliations:** a Department of Rehabilitation Medicine, The Affiliated Jianhu Hospital of Xinglin College, Nantong University, Yancheng, Jiangsu, China.

**Keywords:** Dysphagia, Geriatric Nutritional Risk Index (GNRI), Malnutrition, MIMIC-IV Database, Nutritional Status

## Abstract

Evidence regarding the association between nutritional status and dysphagia in older ICU patients remains limited. We assessed the association between the Geriatric Nutritional Risk Index (GNRI) and the prevalence of dysphagia in this population. Patients aged ≥ 65 years were identified from the MIMIC-IV (v3.1) database and divided into GNRI quartiles: Q1 (≤89.88), Q2 (89.89–99.19), Q3 (99.20–109.38), and Q4 (≥109.39). Associations were examined using logistic regression models (univariable and multivariable). Potential nonlinearity was modeled by restricted cubic splines (RCS), and the inflection point was identified via threshold-effect analysis. Robustness was then assessed through subgroup and sensitivity analyses. In 1576 patients (mean age 77.3 ± 7.8 years; 44.5% female), a lower GNRI was associated with a higher prevalence of dysphagia. Relative to the lowest quartile (Q1, reference), the adjusted odds ratios (ORs) for dysphagia in Q2, Q3, and Q4 were 0.45 (95% CI 0.30–0.69), 0.42 (95% CI 0.27–0.66), and 0.34 (95% CI 0.21–0.55), respectively (all *P* < .001; *P* for trend < 0.001). RCS analysis showed a significant nonlinear association between GNRI and dysphagia (*P* for nonlinearity = 0.01), and the inflection point was located at a GNRI of 91.21 (95% CI 90.85–91.56). These associations were consistent across subgroup and sensitivity analyses, although some weighted estimates should be interpreted with caution. In this cohort of older ICU patients who underwent swallowing assessment within 24 hours of admission, a lower GNRI was associated with a higher prevalence of dysphagia defined by bedside nursing swallow-screening. The association appeared more pronounced within the lower GNRI range and attenuated at higher GNRI levels. Overall, these descriptive findings indicate that dysphagia is more prevalent among patients with a lower GNRI, underscoring the importance of closely monitoring swallowing status in this subgroup.

## 1. Introduction

Malnutrition is a pervasive clinical challenge in hospitalized patients, affecting up to 30% of this population and independently predicting poor outcomes such as longer hospital stays, more severe infection, and higher mortality.^[1–4]^ For critically ill older adults, this risk is amplified by the natural decline in physiological function. Nutritional assessment in this group is important but difficult; the GNRI is widely applied to evaluate nutritional risk in critically ill patients.^[5,6]^ Calculated solely from serum albumin, height, and body weight, the GNRI functions as a simple, objective screening tool that reliably identifies nutritional risk without requiring patient cooperation.^[7–9]^

Dysphagia can lead to aspiration pneumonia, dehydration, delirium, malnutrition, and increased mortality.^[10–13]^ Given the clinical importance of swallowing, increasing attention has been directed to swallowing status in older adults, with numerous studies evaluating prognostic factors in this population.^[14,15]^ Although assessment protocols including bedside screening and instrumental examinations are well established,^[16,17]^ dysphagia is still common but often overlooked among older adults hospitalized in the intensive care unit (ICU).^[18]^ In this high-stakes environment, immediate life-sustaining interventions naturally take precedence upon admission; consequently, the assessment of baseline swallowing function is often overshadowed. This oversight is particularly concerning, as unrecognized dysphagia may hinder recovery. Failure to identify swallowing impairment early in the ICU stay can exacerbate nutritional decline and compromise long-term prognosis.

With advancing age, dysphagia and malnutrition frequently coexist. This relationship is biologically plausible, as malnutrition contributes to systemic muscle wasting that can extend to the swallowing musculature.^[19,20]^ Consequently, nutritional status may be associated with swallowing function. However, the relationship between GNRI‑defined nutritional status and swallowing function among critically ill older adults remains insufficiently characterized. We hypothesized that older ICU patients with a lower GNRI would have a higher prevalence of dysphagia; accordingly, we aimed to quantify this association.

## 2. Methods

### 2.1. Data sources

Data were obtained from MIMIC-IV (v3.1), a public database containing medical and nursing records for ICU patients from 2008 to 2022.^[21]^ The studies involving human subjects were approved by the Ethics Committee of Beth Israel Deaconess Medical Center. All data were anonymized to safeguard patient privacy; therefore, the requirement for informed consent was waived. The database was accessed once the required certification was obtained (Record ID: 54909339). This study followed the STROBE guideline and the principles of the Declaration of Helsinki.^[22]^

### 2.2. Study population

This was a cross-sectional analysis examining the association between baseline nutritional status and dysphagia prevalence among older ICU patients, using data collected within 24 hours of ICU admission. The primary outcome was dysphagia prevalence, and the primary exposure was nutritional status assessed by the GNRI. Inclusion criteria were age ≥ 65 years and first ICU admission (for patients with multiple admissions, only the first was included). Exclusion criteria were: missing height, weight, or serum albumin at ICU admission; no documented swallowing assessment within 24 hours of ICU admission; and ICU length of stay < 24 hours. The GNRI was calculated using established methods^[23–25]^: GNRI = (1.489 × 10 × serum albumin [g/dL]) + (41.7 × (weight [kg]/ ideal body weight [kg])). Ideal body weight (kg) was calculated as follows: Male: 0.75 × height [cm] − 62.5; Female: 0.60 × height [cm] − 40. In our cohort, 83.2% of patients had a weight/ideal body weight ratio > 1; applying the cap would compress the weight term to a constant for these patients, leaving serum albumin as the sole source of variation and markedly reducing the information content of the index. We therefore retained the uncapped definition for the primary analysis and used the conventional capped definition in sensitivity analyses, in which the weight ratio was truncated at 1.^[7]^

### 2.3. Data collection

Patient baseline characteristics were extracted using PostgreSQL (version 13.3). Medical indicators were collected within the first 24 hours after ICU admission. Demographic variables included sex, age, race, height, and weight at admission. Comorbidities assessed were hypertension, diabetes, myocardial infarction, congestive heart failure, chronic pulmonary disease, cerebrovascular disease, sepsis, renal disease, liver disease, dementia, and malignant cancer. Laboratory tests included white blood cell count, hemoglobin level, platelet count, sodium, potassium, calcium, blood urea nitrogen (BUN), creatinine, and albumin level. Scoring systems were recorded: Glasgow Coma Scale (GCS), Admission Acute Physiology Score III (APS III), and Simplified Acute Physiology Score II (SAPS II). Follow-up data included 30-, 365-day, ICU, and hospital mortality, as well as ICU and hospital length of stay.

### 2.4. Assessment of dysphagia

Assessment of dysphagia was performed within 24 hours of ICU admission and was defined as the primary outcome. Nurses conducted an initial swallowing screening, including the Oropharyngeal Catheter score, and assessed patient alertness, ability to remain upright, oropharyngeal secretions, and respiratory stability. Past medical history was reviewed for evidence of dysphagia or the need for a thickened liquid diet. If any of these assessments were positive, a nil per os (nothing by mouth, NPO) order was initiated until specialist consultation. Patients with all negative findings proceeded to a water swallow screening. A positive result (indicating dysphagia) was defined as failure at any stage of the screening. Previous studies indicate that bedside swallowing assessments conducted by nurses offer substantial clinical value.^[11,12,26]^ In the MIMIC-IV database, a swallowing-screening result was documented only when a formal nursing swallow-screen note was entered during the ICU stay, and such screening required the patient to be conscious, alert, and able to cooperate. Consistent with prior MIMIC-IV-based studies,^[13]^ dysphagia was operationalized using the documented bedside nursing swallowing-screening result (itemid = 225118), reflecting a screening-based classification rather than instrumental confirmation (e.g., videofluoroscopic swallow study [VFSS] or fiberoptic endoscopic evaluation of swallowing [FEES]). The recorded values of itemid 225118 were binary: a value of 1 (positive screen) was classified as dysphagia, and a value of 0 (negative screen) as no dysphagia; no indeterminate or incomplete values were recorded. Patients without a documented 0/1 value within the first 24 hours after ICU admission were classified as not having a documented swallowing assessment and were therefore excluded from the outcome analysis. When multiple swallowing screens were recorded for the same patient, the first assessment after ICU admission (earliest charttime) was used to capture admission dysphagia status. Detailed statistics on the distribution of swallowing-assessment counts and timing, including the median time to the first assessment and the consistency of repeated assessments, are summarized (Table S1, Supplemental Digital Content 1).

### 2.5. Statistical analysis

We report normally distributed continuous variables as mean ± SD and skewed variables as median (interquartile range), and we present categorical variables as frequencies (%). Differences across GNRI quartiles were compared using the chi-square test, one-way ANOVA, or the Kruskal–Wallis *H* test, as appropriate for each variable type.

We used univariable and multivariable logistic regression models to examine the association between GNRI and dysphagia. GNRI was analyzed both as a categorical variable and as a continuous variable. Covariates were retained as confounders if they met any of the following: clinical relevance; a univariable association with the outcome (*P* < .10) (Table S2, Supplemental Digital Content 2); or a change of more than 10% in the primary exposure effect estimate after adjustment. Multicollinearity was assessed via the variance inflation factor (VIF); variables with VIF ≥ 5 were excluded from the final model (Table S3, Supplemental Digital Content 3). Missing covariates were imputed using multiple imputation by chained equations (5 imputations) to maintain statistical power and reduce bias; missingness is summarized in Table S4, Supplemental Digital Content 4.

Four nested logistic regression models were specified. In Model 1, no covariates were included. Model 2 incorporated age, sex, and race. Model 3 additionally included white blood cell, hemoglobin, platelet, sodium, calcium, hypertension, diabetes, myocardial infarction, congestive heart failure, dementia, cerebrovascular disease, sepsis, liver disease, and malignant cancer. Model 4 included all variables in Model 3 plus SAPS II. To assess potential nonlinear relationships and threshold effects, restricted cubic splines (RCS) with 4 knots (at the 5th, 35th, 65th, and 95th percentiles) were used. The threshold was estimated using a smooth two-piecewise logistic regression model (threshold effect analysis), with the inflection point determined by the likelihood-ratio test and bootstrap resampling. Subgroup analyses were carried out for age (65–75 vs ≥75 years), sex (female vs male), the presence of dementia, cerebrovascular disease, malignant cancer, or sepsis (no vs yes), and SAPS II (<45 vs ≥ 45). Interaction was assessed using *P* values for interaction, and subgroup results were displayed in forest plots.

Sensitivity analyses were conducted by repeating the primary analyses in the complete-case dataset and after excluding participants with cerebrovascular disease, malignant cancer, or dementia. In addition, several propensity score methods (adjustment, matching, and weighting) were applied. Participants were dichotomized into 2 groups based on the inflection point identified in the threshold-effect analysis (GNRI = 91.21). Propensity score matching (PSM) was performed using a 1:1 nearest neighbor matching algorithm with a caliper width of 0.2 (on the logit scale). Covariate balance was assessed using standardized mean differences (SMD). Covariates included in Model 4 were used for matching. For weighting, we applied inverse probability of treatment weighting (IPTW), standardized mortality ratio weighting (SMRW), pairwise algorithm (PA), and overlap weighting (OW). Detailed descriptions of the sensitivity analyses, including propensity score adjustment and various weighting methods, are provided in the Supplementary Methods. Additionally, we performed an analysis of the association using the conventional capped GNRI definition.

All analyses were conducted with R Statistical Software (version 4.2.2) and the Free Statistics analysis platform (version 2.1, Beijing, China). Statistical significance was defined as a two-sided *P* < .05.

## 3. Results

65,366 patients with a first ICU admission were initially identified in the MIMIC-IV database. We first excluded 29,866 patients aged < 65 years and 6803 patients with an ICU length of stay < 24 hours. Among the remaining 28,697 older patients, 24,861 were excluded due to missing data required for GNRI calculation (missing serum albumin: n = 21,289; height: n = 3537; or weight: n = 35). Subsequently, 2260 patients without a documented swallowing assessment were excluded. Finally, 1576 older ICU patients were included (Fig. 1). Table S5, Supplemental Digital Content 5 presents the baseline characteristics and clinical outcomes of patients with and without a documented swallowing assessment.

**Figure 1.**
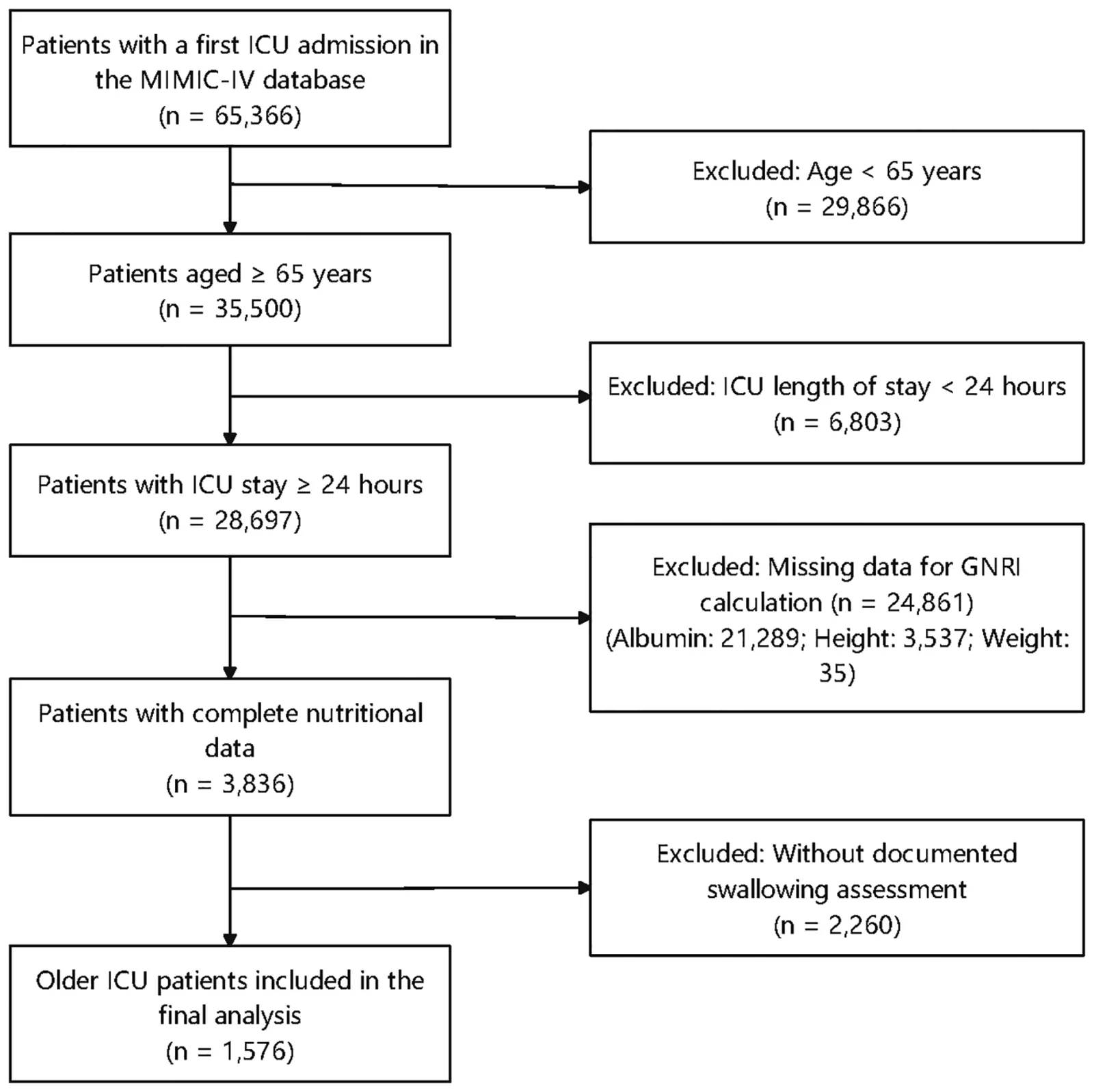
A flow chart of study patients. GNRI = Geriatric Nutritional Risk Index, ICU = intensive care unit, MIMIC-IV = Medical Information Mart for Intensive Care IV.

### 3.1. Baseline characteristics

Among 1576 participants (mean age 77.3 ± 7.8 years), 44.5% (n = 701) were female. Baseline characteristics by GNRI quartile (Q1 ≤ 89.88, Q2 89.89–99.19, Q3 99.20–109.38, and Q4 ≥ 109.39) are presented in Table 1. Higher GNRI quartiles were associated with younger age and greater body weight. These groups also showed higher hemoglobin, albumin, and calcium levels, and a higher prevalence of diabetes and congestive heart failure. Conversely, sepsis and malignant cancer were less frequent, and SAPS II scores were significantly lower. Dysphagia was likewise less common at higher GNRI levels (9.4% in Q4 vs 23.6% in Q1). Consistently, mortality decreased with increasing GNRI: hospital mortality was lower in Q4 than in Q1 (12.4 vs 27.9%), as was ICU mortality. Moreover, mortality at 30 and 365 days was reduced in Q4 compared with Q1 (15.5 vs 36.3%, and 28.9 vs 59.9%, respectively; all *P* < .001).

**Table 1:** Baseline characteristics and clinical outcomes of the study participants stratified by GNRI quartiles.

Variables	Total (n = 1576)	Q1 (≤89.88) (n = 394)	Q2 (89.89–99.19) (n = 394)	Q3 (99.20–109.38) (n = 394)	Q4 (≥109.39) (n = 394)	*P* value
Age, years	77.3 (7.8)	78.3 (7.9)	78.1 (8.0)	78.0 (7.8)	74.8 (6.8)	<.001
Female, n (%)	701 (44.5)	176 (44.7)	163 (41.4)	166 (42.1)	196 (49.7)	.077
Race, n (%)						.408
White	1113 (70.6)	290 (73.6)	278 (70.6)	264 (67.0)	281 (71.3)	
Black/African American	102 (6.5)	25 (6.3)	21 (5.3)	32 (8.1)	24 (6.1)	
Other	361 (22.9)	79 (20.1)	95 (24.1)	98 (24.9)	89 (22.6)	
Height, cm	167.5 (10.7)	167.0 (10.5)	168.5 (10.2)	167.5 (10.2)	167.1 (11.8)	.208
Weight, kg	79.0 (21.8)	62.9 (13.9)	73.0 (13.8)	79.9 (13.6)	100.1 (24.3)	<.001
GNRI	100.2 (16.8)	80.7 (7.6)	94.6 (2.6)	104.2 (2.8)	121.5 (13.1)	<.001
GCS at admission	12.3 (3.7)	11.8 (3.9)	12.2 (3.8)	12.7 (3.3)	12.5 (3.7)	.003
SAPS II	41.8 (13.1)	46.5 (14.1)	42.2 (12.2)	39.3 (11.8)	39.1 (12.8)	<.001
WBC, ×10^9^/L	9.3 (6.8, 12.8)	9.8 (6.6, 14.6)	9.3 (6.5, 12.8)	9.6 (7.0, 12.4)	8.7 (7.0, 11.8)	.156
Hemoglobin, g/dL	10.1 (2.2)	9.3 (2.0)	9.7 (1.9)	10.5 (2.3)	10.8 (2.3)	<.001
Platelet, ×10^9^/L	188.7 (102.0)	187.0 (122.2)	193.9 (104.0)	188.3 (94.5)	185.8 (83.8)	.695
Sodium, mmol/L	136.3 (5.7)	136.4 (6.6)	136.2 (5.5)	136.2 (5.7)	136.5 (4.7)	.886
Calcium, mg/dL	8.1 (0.8)	7.6 (0.9)	8.1 (0.8)	8.2 (0.8)	8.4 (0.8)	<.001
Albumin, g/dL	3.2 (0.6)	2.6 (0.5)	3.1 (0.4)	3.4 (0.5)	3.6 (0.5)	<.001
**Comorbidities, n (%**)						
Hypertension	602 (38.2)	136 (34.5)	142 (36.0)	172 (43.7)	152 (38.6)	.045
Diabetes	528 (33.5)	85 (21.6)	130 (33.0)	140 (35.5)	173 (43.9)	<.001
Myocardial infarction	437 (27.7)	71 (18.0)	114 (28.9)	137 (34.8)	115 (29.2)	<.001
Congestive heart failure	658 (41.8)	133 (33.8)	179 (45.4)	162 (41.1)	184 (46.7)	<.001
Cerebrovascular disease	232 (14.7)	46 (11.7)	57 (14.5)	63 (16.0)	66 (16.8)	.193
Sepsis	995 (63.1)	290 (73.6)	248 (62.9)	232 (58.9)	225 (57.1)	<.001
Liver disease	235 (14.9)	68 (17.3)	56 (14.2)	52 (13.2)	59 (15.0)	.428
Malignant cancer	264 (16.8)	101 (25.6)	75 (19.0)	49 (12.4)	39 (9.9)	<.001
Dementia	68 (4.3)	24 (6.1)	22 (5.6)	12 (3.0)	10 (2.5)	.028
**Outcomes**						
Dysphagia, n (%)	222 (14.1)	93 (23.6)	49 (12.4)	43 (10.9)	37 (9.4)	<.001
ICU length of stay, days	3.1 (1.9, 6.0)	3.3 (2.0, 6.0)	3.5 (1.9, 6.8)	3.0 (1.9, 5.2)	3.1 (1.9, 6.1)	.381
Hospital length of stay, days	8.2 (5.1, 14.2)	9.0 (5.4, 16.8)	8.8 (5.7, 15.4)	7.5 (5.0, 12.6)	7.8 (4.9, 13.2)	.011
ICU mortality, n (%)	212 (13.5)	80 (20.3)	47 (11.9)	42 (10.7)	43 (10.9)	<.001
Hospital mortality, n (%)	274 (17.4)	110 (27.9)	60 (15.2)	55 (14.0)	49 (12.4)	<.001
30-day mortality, n (%)	349 (22.1)	143 (36.3)	77 (19.5)	68 (17.3)	61 (15.5)	<.001
365-day mortality, n (%)	613 (38.9)	236 (59.9)	142 (36.0)	121 (30.7)	114 (28.9)	<.001

Data are presented as mean (standard deviation) for normally distributed continuous variables, median (interquartile range) for skewed continuous variables, and number (percentage) for categorical variables. GNRI = Geriatric Nutritional Risk Index, SAPS II = Simplified Acute Physiology Score II, WBC = white blood cell. *P* values were calculated using one-way ANOVA, Kruskal-Wallis test, or Chi-square test, as appropriate.

### 3.2. Association between GNRI and dysphagia

In multivariable logistic regression (Table 2), GNRI was inversely associated with the odds of dysphagia; in the fully adjusted model (Model 4), each 1-unit increase corresponded to a 3% lower odds (OR 0.97; 95% CI 0.96–0.98; *P* < .001). When GNRI was stratified into quartiles, Q1 (≤89.88) served as the reference group, while the adjusted ORs for dysphagia in Q2, Q3, and Q4 were 0.45 (95% CI 0.30–0.69), 0.42 (95% CI 0.27–0.66), and 0.34 (95% CI 0.21–0.55), respectively (all *P* < .001; *P* for trend < .001).

**Table 2:** Multivariable logistic regression for GNRI and dysphagia.

Variable	n (total)	n (%)	Model 1		Model 2		Model 3		Model 4	
			OR (95% CI)	*P* Value	OR (95% CI)	*P* Value	OR (95% CI)	*P* Value	OR (95% CI)	*P* Value
GNRI	1576	222 (14.1)	0.97 (0.96–0.98)	<.001	0.97 (0.96–0.98)	<.001	0.97 (0.95–0.98)	<.001	0.97 (0.96–0.98)	<.001
Quartiles										
Q1 (≤89.88)	394	93 (23.6)	1 (Ref)		1 (Ref)		1 (Ref)		1 (Ref)	
Q2 (89.89–99.19)	394	49 (12.4)	0.46 (0.31–0.67)	<.001	0.47 (0.32–0.68)	<.001	0.43 (0.29–0.65)	<.001	0.45 (0.30–0.69)	<.001
Q3 (99.20–109.38)	394	43 (10.9)	0.40 (0.27–0.59)	<.001	0.40 (0.27–0.59)	<.001	0.39 (0.25–0.61)	<.001	0.42 (0.27–0.66)	<.001
Q4 (≥109.39)	394	37 (9.4)	0.34 (0.22–0.51)	<.001	0.34 (0.23–0.52)	<.001	0.32 (0.20–0.52)	<.001	0.34 (0.21–0.55)	<.001
Trend test	1576	222 (14.1)		<.001		<.001		<.001		<.001

Model 1: adjusted for none.

Model 2: adjusted for age, sex, and race.

Model 3: adjusted for model 2 + white blood cell, hemoglobin, platelet, sodium, calcium, hypertension, diabetes, myocardial infarction, congestive heart failure, dementia, cerebrovascular disease, sepsis, liver disease, and malignant cancer.

Model 4: adjusted for model 3 + SAPS II.

GNRI = Geriatric Nutritional Risk Index, OR = odds ratio, SAPS II = Simplified Acute Physiology Score II.

### 3.3. Nonlinear association and threshold effects of GNRI and dysphagia

RCS analysis revealed a nonlinear association between GNRI and the odds of dysphagia (*P* for overall < .001, *P* for nonlinearity = .01; Fig. 2). The threshold-effect analysis identified an inflection point of 91.21 (95% CI 90.85–91.56). Below this inflection point (GNRI < 91.21), each 1-unit increase in GNRI was associated with a 5% reduction in the odds of dysphagia (OR 0.95; 95% CI 0.92–0.98; *P <* .001). However, at or above this threshold (GNRI ≥ 91.21), although the visual trend in the RCS plot suggested a continued decline in risk, the association was attenuated and did not reach statistical significance in the threshold effect analysis (adjusted OR 0.99; 95% CI 0.98–1.01; *P =* .236) (Table 3).

**Table 3:** Threshold effect analysis of the relationship between GNRI and dysphagia.

GNRI Range	Adjusted OR (95% CI)	*P* value
<91.21	0.95 (0.92–0.98)	<.001
≥91.21	0.99 (0.98–1.01)	.236
Likelihood Ratio test		.009

Data were adjusted for age, sex, race, white blood cell, hemoglobin, platelet, sodium, calcium, hypertension, diabetes, myocardial infarction, congestive heart failure, dementia, cerebrovascular disease, sepsis, liver disease, malignant cancer, and Simplified Acute Physiology Score II.

GNRI = Geriatric Nutritional Risk Index, OR = odds ratio.

**Figure 2.**
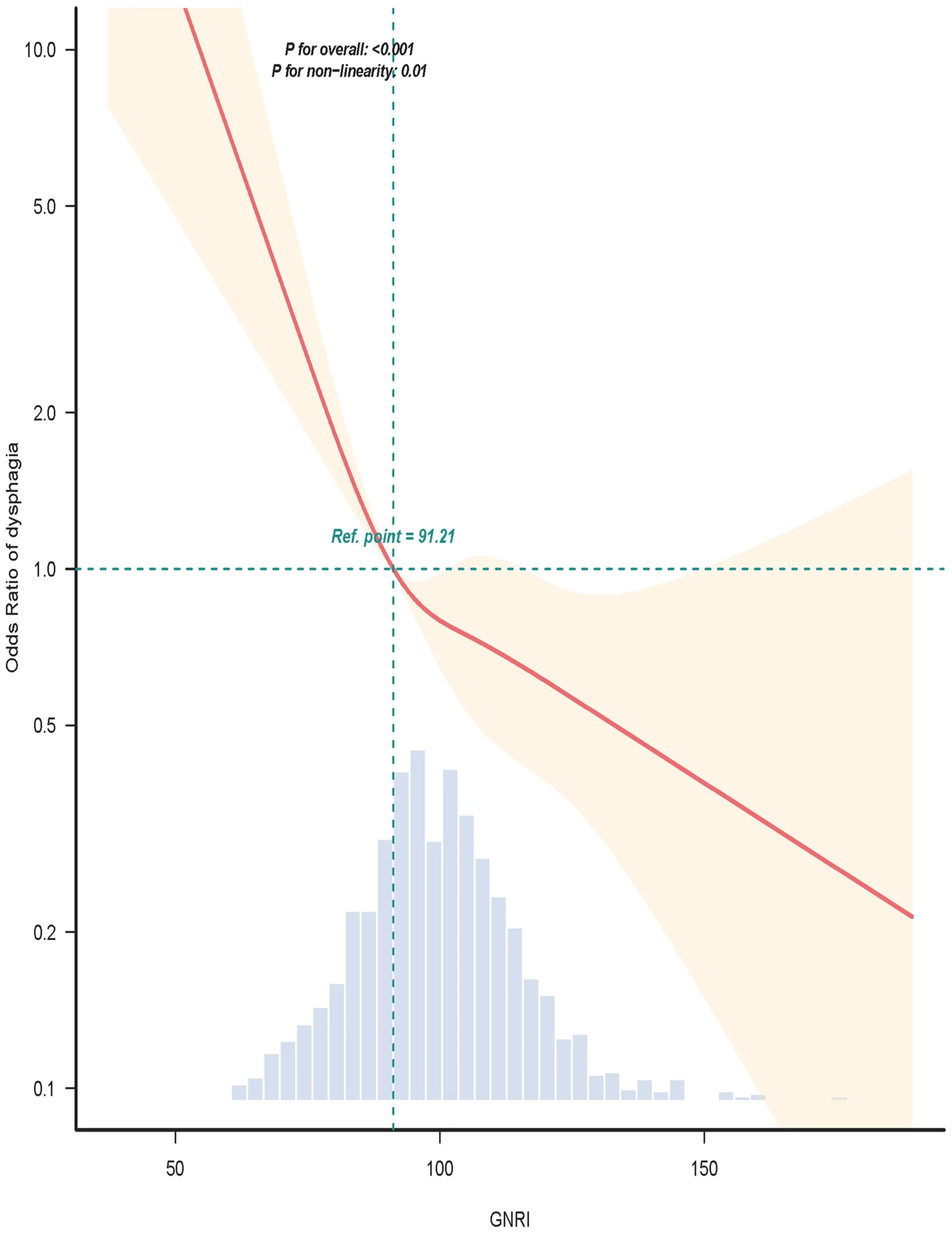
Association between GNRI and dysphagia odds ratio. The solid red line represents the fitted curve, with the shaded area denoting 95% confidence intervals. The reference value was set at the estimated inflection point (GNRI = 91.21); this choice does not affect the curve shape or the *P* value for nonlinearity. Data were adjusted for age, sex, race, white blood cell, hemoglobin, platelet, sodium, calcium, hypertension, diabetes, myocardial infarction, congestive heart failure, dementia, cerebrovascular disease, sepsis, liver disease, malignant cancer, and Simplified Acute Physiology Score II. GNRI = Geriatric Nutritional Risk Index.

### 3.4. Subgroup analysis

In subgroup analyses (Fig. 3), the inverse association between GNRI and dysphagia remained generally consistent across strata. No significant interactions were observed between GNRI and any of the stratification variables (all *P* for interaction > 0.05). While the associations in the dementia and malignant cancer subgroups were not statistically significant (OR 0.99 [0.93–1.06] and OR 0.98 [0.96–1.01], respectively), the direction of the association was consistent with the overall trend.

**Figure 3.**
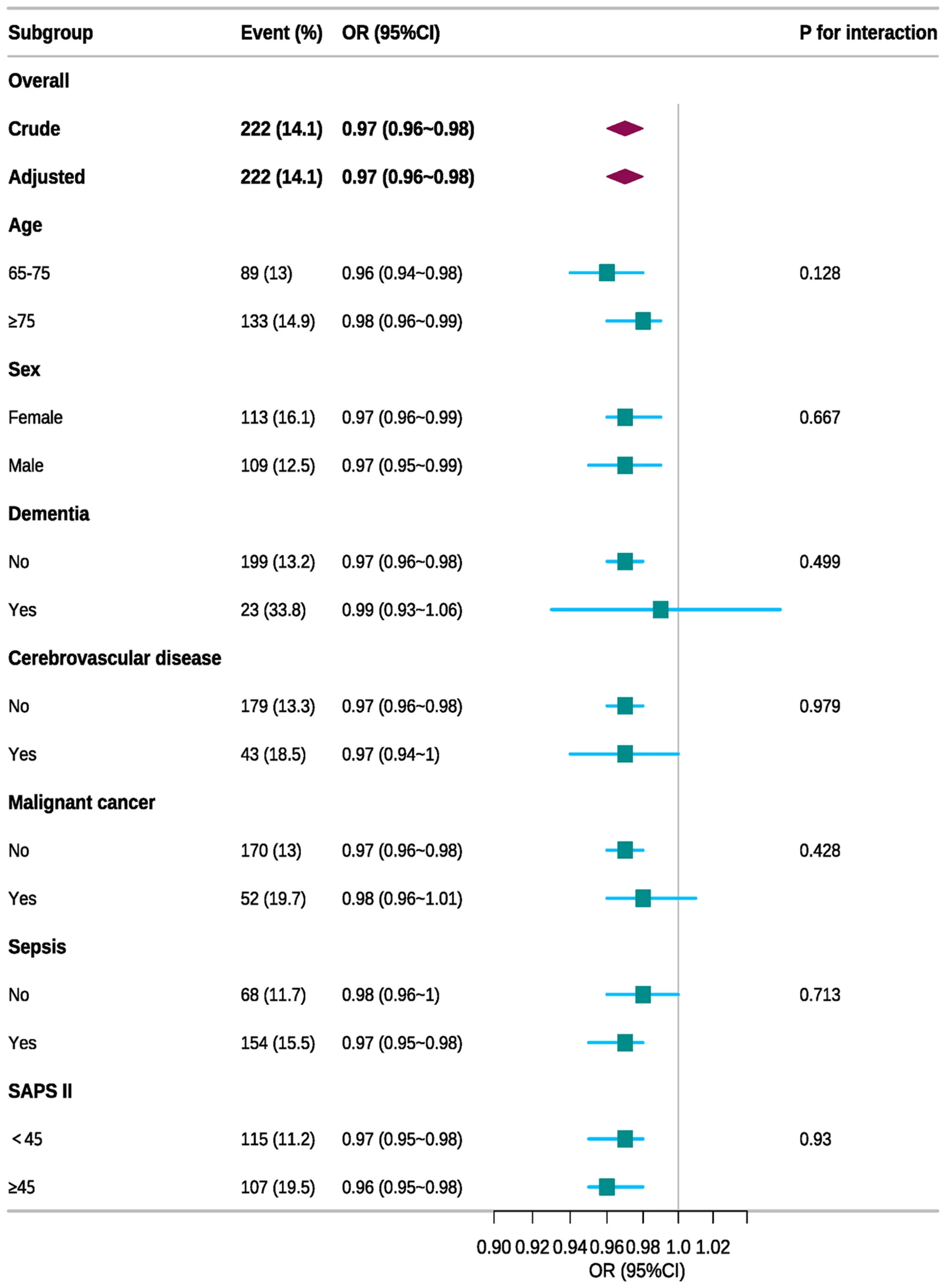
Subgroup analyses of the association between GNRI and dysphagia according to basic features. Data were adjusted for age, sex, race, white blood cell, hemoglobin, platelet, sodium, calcium, hypertension, diabetes, myocardial infarction, congestive heart failure, dementia, cerebrovascular disease, sepsis, liver disease, malignant cancer, and Simplified Acute Physiology Score II. GNRI = Geriatric Nutritional Risk Index.

### 3.5. Sensitivity analysis

Several sensitivity analyses were performed. First, we repeated the primary analysis using the original dataset without multiple imputation (Table S6, Supplemental Digital Content 6). Second, we excluded participants with specific comorbidities that influence swallowing function, including cerebrovascular disease, malignant cancer, or dementia (Table S7, Supplemental Digital Content 7). Furthermore, to further account for measured confounding, we performed several propensity score methods (adjustment, matching, and weighting). Participants were dichotomized into 2 groups based on a GNRI threshold of 91.21 (<91.21 vs ≥91.21). Across these methods, the results remained consistent (Fig. 4). However, extreme weights were present in the IPTW and SMRW analyses, so those two estimates should be interpreted with caution. Detailed descriptions of each sensitivity-analysis approach, including the matched sample sizes or sums of weights, dysphagia event counts, covariate balance, and effect estimates, are provided in the Supplementary Methods and summarized in Tables S8 and S9, Supplemental Digital Content 8 and Figure S1, Supplemental Digital Content 9. Additionally, we performed an analysis using the conventional capped GNRI definition; the inverse association with dysphagia remained consistent (continuous OR 0.95, 95% CI 0.93–0.96; quartile ORs ranging from 0.43 to 0.33) (Table S10, Supplemental Digital Content 10). Collectively, the inverse association between GNRI and dysphagia was consistent across the sensitivity analyses, although the IPTW and SMRW estimates should be interpreted with caution.

**Figure 4.**
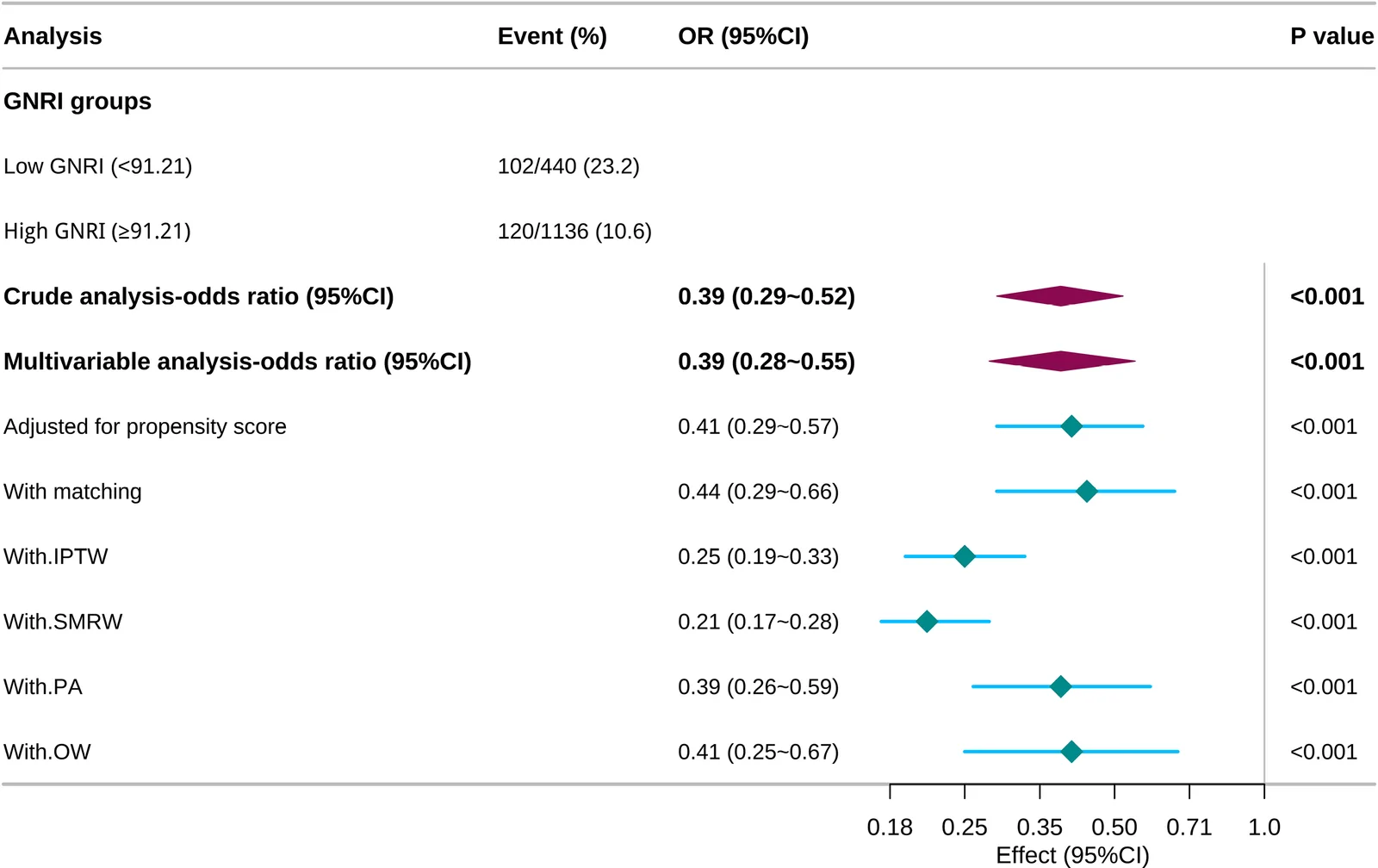
Sensitivity analyses for the association between GNRI categories and dysphagia using varying propensity score models. The plot displays ORs comparing high (≥91.21) vs. low (<91.21) GNRI groups (Reference: Low GNRI). CI = confidence interval, GNRI = geriatric nutritional risk index, IPTW = inverse probability of treatment weighting, OR = odds ratio, OW = Overlap Weighting, PA = Pairwise Algorithm, SMRW = standardized mortality ratio weighting.

## 4. Discussion

In this study of older ICU patients, we identified an inverse association between nutritional status (assessed by GNRI) and the prevalence of dysphagia defined by bedside nursing swallow-screening. We found a nonlinear association between GNRI and dysphagia. Our subgroup and sensitivity analyses supported the robustness of the observed inverse association, although some weighted estimates should be interpreted with caution. Furthermore, consistent with previous literature, low GNRI was associated with higher short- and long-term mortality in this cohort.

Our findings align with established evidence linking malnutrition to adverse outcomes, including mortality, delirium, and prolonged hospital stay.^[12,27–30]^ This deleterious impact of low nutritional status extends beyond critical care settings. Notably, among patients with hypertension, individuals in the lowest GNRI tertile exhibited a markedly higher risk of kidney events (HR 3.59, 95% CI: 1.96–6.63), all-cause death, and cardiovascular events compared with those in the highest tertile.^[31]^ However, our study extends current knowledge by quantifying the nonlinear association between GNRI and dysphagia. While previous studies have described a U-shaped relationship between GNRI and the occurrence of sepsis,^[32]^ we observed an inverse association specifically at lower GNRI levels. We identified an inflection point at 91.21 (95% CI 90.85–91.56), below which higher nutritional status was associated with a reduced likelihood of dysphagia. Conversely, this association was attenuated above this threshold. This suggests that the vulnerability to dysphagia is concentrated primarily within the malnutrition spectrum. Therefore, a GNRI below 91.21 (95% CI 90.85–91.56) may be considered an exploratory indicator of a higher prevalence of screening-defined dysphagia in our cohort; however, since this cutoff was derived from a single dataset, it requires external validation before any clinical application.

Dysphagia represents a complex pathophysiological process.^[33,34]^ The observed association is biologically plausible and may be mediated through several mechanisms. First, it aligns with the concept of “sarcopenic dysphagia,” where malnutrition may contribute to generalized skeletal muscle atrophy and reduced protein synthesis.^[35]^ Such systemic loss of muscle mass may offer one plausible pathway through which nutritional depletion is associated with dysphagia. Second, malnutrition is often accompanied by a chronic inflammatory state. As indicated by our data, the lowest GNRI quartile (Q1) exhibited a significantly higher prevalence of sepsis compared with the highest quartile (Q4) (73.6 vs 57.1%, *P <* .001). Systemic inflammation may alter metabolic pathways and impair neuromuscular function, indirectly harming deglutition muscles and nerve centers.^[36]^ Finally, adequate nutritional status is crucial for neurological recovery. In older ICU patients who often suffer from concurrent cerebrovascular or neurological insults, malnutrition may impede neuronal repair and aggravate central functional impairment, further compromising the precise neuromodulation required for the oropharyngeal phase of swallowing.^[37,38]^ Taken together, sarcopenic dysphagia, systemic inflammation, and impaired neurological recovery provide plausible explanations that warrant prospective confirmation. Importantly, reciprocal processes are also possible: dysphagia can reduce oral intake, thereby worsening nutritional status and lowering GNRI.

Because both GNRI and dysphagia were assessed at the same time point, the cross-sectional design cannot establish the temporal sequence, and reverse causality cannot be fully excluded. Therefore, the findings should be interpreted as a balanced association that may reflect potentially bidirectional relationships, rather than evidence that low GNRI causes dysphagia. We also note that GNRI may partially reflect the severity of underlying disease and the overall catabolic state, rather than an independent determinant of swallowing function. Thus, the observed association should be regarded as a signal for risk stratification rather than causal inference.

Our sensitivity and subgroup analyses supported the robustness of the observed inverse association. When a capped GNRI was used in sensitivity analyses (≤1.0 for the weight-to-ideal ratio), the inverse association with dysphagia remained stable. In the dementia and malignant cancer subgroups, the associations were not statistically significant (OR 0.99 [0.93–1.06] and OR 0.98 [0.96–1.01], respectively), but the directions were consistent with the overall trend; these findings should therefore be interpreted with caution, as small sample sizes and few events limited statistical power. Low GNRI often coexists with cerebrovascular disease, dementia, and tumors, and these conditions are known risk factors for dysphagia.^[39–42]^ It is crucial to determine if the observed association comes from these comorbidities. In our sensitivity analyses, we excluded patients with these specific conditions. Even after these exclusions, the inverse association between GNRI and dysphagia remained stable and significant. The persistence of the inverse association after these exclusions is compatible with malnutrition being associated with dysphagia prevalence; however, the cross-sectional design precludes conclusions about causality, and the association may also reflect the burden of underlying disease.

This finding has potential practical value for ICU management. Instrumental swallowing assessments are often difficult to perform on unstable patients. In contrast, GNRI is a simple and objective metric. The inflection point we observed suggests that lower GNRI values (e.g., < 91.21) were associated with a higher prevalence of screening-defined dysphagia in this cohort. Given the observed association between lower GNRI and a higher prevalence of dysphagia in this cohort, swallowing assessment may be particularly relevant in patients with lower GNRI when feasible. Whether GNRI-guided assessment improves detection or patient outcomes warrants prospective validation.

Several limitations should be noted in our study. First, the retrospective design may introduce selection bias and residual confounding, precluding causal inference; reverse causality (dysphagia leading to malnutrition) also cannot be entirely ruled out. Second, patients who were unable to cooperate with the swallowing assessment were excluded, and dysphagia was defined by bedside nursing screening rather than instrumental confirmation (VFSS or FEES), which may lead to misclassification of dysphagia and affect the estimated prevalence. Third, patients were excluded when any GNRI component (serum albumin, height, or weight) was missing at admission; if missingness is informative (e.g., more severely ill patients may be more likely to have incomplete admission laboratory data), this may introduce selection bias. The direction of selection bias related to swallowing assessment is also uncertain: screening may be performed selectively in higher-risk patients (which could overestimate the prevalence), whereas the exclusion of patients too unstable or unconscious to be assessed may underestimate it. Fourth, because the primary analysis used an uncapped GNRI definition, direct comparison of our quartile boundaries and threshold with studies that apply conventional GNRI cutoffs should be made with caution. Finally, we restricted inclusion to patients assessed within 24 hours of admission, potentially excluding those with delayed-onset dysphagia. Our findings therefore apply to older ICU patients sufficiently stable for swallowing assessment within 24 hours of admission. Future prospective studies using objective diagnostic criteria are warranted.

## 5. Conclusion

In this cohort of older ICU patients who underwent swallowing assessment within 24 hours of admission, lower GNRI was associated with a higher prevalence of dysphagia defined by bedside nursing swallow-screening. The association appeared more pronounced within the lower GNRI range and attenuated at higher GNRI levels. Overall, these descriptive findings indicate that dysphagia is more prevalent among patients with lower GNRI, underscoring the importance of closely monitoring swallowing status in this subgroup.

## Acknowledgments

We thank Qilin Yang (The Second Affiliated Hospital of Guangzhou Medical University, Guangzhou, Guangdong, China) for statistical support and for reviewing the manuscript. Qilin Yang has given permission to be named in this Acknowledgments.

## Author contributions

**Conceptualization:** Chong Wang.

**Data curation:** Chong Wang, Lili Lv, Rongrong Ma.

**Formal analysis:** Chong Wang, Rongrong Ma.

**Investigation:** Chong Wang, Rongrong Ma.

**Methodology:** Chong Wang, Rongrong Ma.

**Project administration:** Chong Wang.

**Software:** Chong Wang.

**Supervision:** Chong Wang.

**Writing – review & editing:** Chong Wang.

**Validation:** Lili Lv.

**Visualization:** Lili Lv.

**Writing – original draft:** Lili Lv, Rongrong Ma.






















