# Supplementary material for: Association between the geriatric nutritional risk index and dysphagia in older critically ill patients: A retrospective analysis of the MIMIC-IV database
Source: Medicine (Baltimore). 2026 Nov 6;105(45):e51456. doi: 10.1097/MD.0000000000051456 (PMC13747612; doi:10.1097/MD.0000000000051456)
Supplement: Supplementary file 1 [file medi-105-e51456-s001.docx]

**Supplementary Table 1.** Distribution of swallowing assessments (itemid 225118)

| **Characteristic** | **ICU admissions, n** | **Proportion, %** |
| --- | --- | --- |
| **Swallowing assessment status (n = 94,458 ICU admissions)** | | |
| Total ICU admissions | 94,458 | 100 |
| ICU admissions with any recorded assessment (all time) | 48,617 | 51.5 |
| Documented assessment within 24 h | 47,279 | 50.1 |
| First assessment positive (dysphagia) | 5,783 | 12.2 |
| First assessment negative (no dysphagia) | 41,496 | 87.8 |
| Without a documented assessment within 24 h | 47,179 | 49.9 |
| **Number of swallowing assessments per assessed admission within 24 h (n = 47,279)** | | |
| 1 | 21,160 | 44.8 |
| 2 | 13,735 | 29.1 |
| 3 | 6,744 | 14.3 |
| 4 | 3,084 | 6.5 |
| ≥5 | 2,556 | 5.4 |
| **Time from ICU admission to first assessment (n = 47,279)** | | |
| 0–1 h | 14,151 | 29.9 |
| 1–6 h | 25,123 | 53.1 |
| 6–12 h | 6,550 | 13.9 |
| 12–24 h | 1,455 | 3.1 |
| Median (IQR), hours | 2.03 (0.82–4.48) | – |
| **Repeated assessments within 24 h (n = 47,279)** | | |
| Admissions with >1 assessment | 26,119 | 55.2 |
| First negative → positive by the last assessment | 64 | 0.14 |
| First positive → negative by the last assessment | 45 | 0.10 |

Note: Among all 94,458 ICU admissions in the MIMIC-IV cohort, 47,279 (50.1%) had at least one charted swallowing assessment within 24 hours after ICU admission. The first assessment (earliest charttime, rn = 1) was used to define dysphagia status. Time is measured from ICU admission (intime) to the first assessment (charttime). **Abbreviations:** ICU, intensive care unit; MIMIC-IV, Medical Information Mart for Intensive Care IV; IQR, interquartile range.
