# Supplementary material for: Association between the geriatric nutritional risk index and dysphagia in older critically ill patients: A retrospective analysis of the MIMIC-IV database
Source: Medicine (Baltimore). 2026 Nov 6;105(45):e51456. doi: 10.1097/MD.0000000000051456 (PMC13747612; doi:10.1097/MD.0000000000051456)
Supplement: Supplementary file 2 [file medi-105-e51456-s002.docx]

**Supplementary Table 2.** Univariable logistic regression analysis of factors associated with dysphagia

| Variable | OR (95% CI) | *P* value |
| --- | --- | --- |
| Age, years | 1.02 (1-1.04) | 0.041 |
| Sex |  |  |
| Female | 1(reference) |  |
| Male | 0.74 (0.56-0.98) | 0.038 |
| Race |  |  |
| White | 1(reference) |  |
| Black/African American | 1.20 (0.69–2.07) | 0.514 |
| Other | 0.88 (0.62–1.25) | 0.462 |
| White blood cell, ×10⁹/L | 1.01 (1–1.02) | 0.113 |
| Hemoglobin, g/dL | 0.94 (0.88–1) | 0.119 |
| Platelet, ×10⁹/L | 1.003 (1.001–1.004) | <0.001 |
| Sodium, mmol/L | 1.04 (1.02–1.07) | 0.001 |
| Potassium, mmol/L | 0.97 (0.76–1.23) | 0.795 |
| Calcium, mg/dL | 1.09 (0.92–1.29) | 0.317 |
| Blood urea nitrogen, mg/dL | 1.005 (0.998–1.011) | 0.15 |
| Creatinine, mg/dL | 0.89 (0.77–1.03) | 0.117 |
| Glucose | 0.999 (0.996–1.003) | 0.648 |
| Hypertension |  |  |
| No | 1(reference) |  |
| Yes | 0.96 (0.72–1.29) | 0.789 |
| Diabetes |  |  |
| No | 1(reference) |  |
| Yes | 1.06 (0.79–1.43) | 0.687 |
| Dementia |  |  |
| No | 1(reference) |  |
| Yes | 3.36 (1.99–5.68) | <0.001 |
| Myocardial infarction |  |  |
| No | 1(reference) |  |
| Yes | 0.57 (0.4–0.81) | 0.002 |
| Congestive heart failure |  |  |
| No | 1(reference) |  |
| Yes | 0.76 (0.56–1.02) | 0.063 |
| Chronic pulmonary disease |  |  |
| No | 1(reference) |  |
| Yes | 0.88 (0.64–1.21) | 0.423 |
| Cerebrovascular disease, |  |  |
| No | 1(reference) |  |
| Yes | 1.48 (1.03–2.14) | 0.036 |
| Sepsis |  |  |
| No | 1(reference) |  |
| Yes | 1.38 (1.02–1.88) | 0.038 |
| Renal disease |  |  |
| No | 1(reference) |  |
| Yes | 0.78 (0.57–1.08) | 0.14 |
| Liver disease |  |  |
| No | 1(reference) |  |
| Yes | 0.56 (0.35–0.89) | 0.015 |
| Malignant cancer |  |  |
| No | 1(reference) |  |
| Yes | 1.65 (1.17–2.32) | 0.004 |
| Acute Physiology Score III | 1.009 (1.004–1.015) | <0.001 |
| Simplified Acute Physiology Score II | 1.02 (1.01–1.03) | <0.001 |

**Abbreviations:** OR, odds ratio; CI, confidence interval;
