# Supplementary material for: Association between the geriatric nutritional risk index and dysphagia in older critically ill patients: A retrospective analysis of the MIMIC-IV database
Source: Medicine (Baltimore). 2026 Nov 6;105(45):e51456. doi: 10.1097/MD.0000000000051456 (PMC13747612; doi:10.1097/MD.0000000000051456)
Supplement: Supplementary file 3 [file medi-105-e51456-s003.docx]

**Supplementary Table 3. Results of covariate selection based on effect size change (>10%) and multicollinearity check (VIF < 5)**

| Term1 | Change.percentage1 | Change.percentage2 | GVIF | DF | GVIF^(1/(2*Df)) | colinearity | select |
| --- | --- | --- | --- | --- | --- | --- | --- |
| Crude | Ref. | Ref. | 1.43 | 1 | 1.196 | 0 | Ref. |
| Age | -2 | -3.3 | 1.333 | 1 | 1.154 | 0 | No |
| Gender | 0.9 | -4.3 | 1.173 | 1 | 1.083 | 0 | No |
| Race | -0.2 | 0.9 | 1.159 | 2 | 1.038 | 0 | No |
| WBC | -1.1 | 0 | 1.151 | 1 | 1.073 | 0 | No |
| Hemoglobin | -1.8 | 3.1 | 1.302 | 1 | 1.141 | 0 | No |
| Platelets_min | -1.5 | 5.6 | 1.162 | 1 | 1.078 | 0 | No |
| Sodium | -1 | -1.3 | 2.963 | 1 | 1.721 | 0 | No |
| Potassium | 0.1 | 0 | 1.323 | 1 | 1.15 | 0 | No |
| Calcium | 19.8 | -11.2 | 1.542 | 1 | 1.242 | 0 | Yes |
| BUN | -0.8 | 1.4 | 2.505 | 1 | 1.583 | 0 | No |
| Creatinine | -0.3 | 2.8 | 2.326 | 1 | 1.525 | 0 | No |
| Glucose | 2 | 2.3 | 1.176 | 1 | 1.084 | 0 | No |
| Hypertension | 0.1 | -1.2 | 1.167 | 1 | 1.08 | 0 | No |
| Diabetes | 5 | -3 | 1.257 | 1 | 1.121 | 0 | No |
| Myocardial infarction | -4.3 | 1.5 | 1.107 | 1 | 1.052 | 0 | No |
| Congestive heart failure | -2.1 | 0.6 | 1.302 | 1 | 1.141 | 0 | No |
| Chronic pulmonary disease | 0.6 | -1.2 | 1.089 | 1 | 1.044 | 0 | No |
| Cerebrovascular disease | 1.4 | 1.2 | 1.159 | 1 | 1.076 | 0 | No |
| Dementia | -3.8 | 2.6 | 1.125 | 1 | 1.061 | 0 | No |
| Sepsis | -2.7 | 0 | 1.272 | 1 | 1.128 | 0 | No |
| Renal disease | -0.2 | -0.1 | 1.396 | 1 | 1.182 | 0 | No |
| Liver_disease | 1.6 | 0.8 | 1.126 | 1 | 1.061 | 0 | No |
| Malignant cancer | -4 | 1.7 | 1.234 | 1 | 1.111 | 0 | No |
| APS III | -7.9 | -1.6 | 5.545 | 1 | 2.355 | 1 | No (collinearity) |
| SAPS II | -9.1 | -0.2 | 2.909 | 1 | 1.706 | 0 | No |

**Abbreviations:** WBC, white blood cell; BUN, blood urea nitrogen; APS III, Acute Physiology Score III; SAPS II, Simplified Acute Physiology Score II.
