# Supplementary material for: Association between the geriatric nutritional risk index and dysphagia in older critically ill patients: A retrospective analysis of the MIMIC-IV database
Source: Medicine (Baltimore). 2026 Nov 6;105(45):e51456. doi: 10.1097/MD.0000000000051456 (PMC13747612; doi:10.1097/MD.0000000000051456)
Supplement: Supplementary file 4 [file medi-105-e51456-s004.docx]

**Supplementary Table 4.** Summary of missing data by variable in the study population

| Variable | Number of missing | Missing Percentage (%) |
| --- | --- | --- |
| Calcium | 48 | 3.05 |
| Hemoglobin | 1 | 0.06 |
