# Supplementary material for: Association between the geriatric nutritional risk index and dysphagia in older critically ill patients: A retrospective analysis of the MIMIC-IV database
Source: Medicine (Baltimore). 2026 Nov 6;105(45):e51456. doi: 10.1097/MD.0000000000051456 (PMC13747612; doi:10.1097/MD.0000000000051456)
Supplement: Supplementary file 5 [file medi-105-e51456-s005.docx]

**Supplementary Table 5.** Baseline characteristics and clinical outcomes of patients with and without a documented swallowing assessment

| Variables | Total (n = 3836) | Swallowing assessment (n = 1576) | No swallowing assessment (n = 2260) | *P* value |
| --- | --- | --- | --- | --- |
| Age, years | 76.9 (7.8) | 77.3 (7.8) | 76.6 (7.8) | 0.008 |
| Female, n (%) | 1713 (44.7) | 701 (44.5) | 1012 (44.8) | 0.855 |
| Race, n (%) |  |  |  | < 0.001 |
| White | 2469 (64.4) | 1113 (70.6) | 1356 (60.0) |  |
| Black/African American | 285 (7.4) | 102 (6.5) | 183 (8.1) |  |
| Other | 1082 (28.2) | 361 (22.9) | 721 (31.9) |  |
| Height, cm | 167.6 (10.5) | 167.5 (10.7) | 167.6 (10.4) | 0.776 |
| Weight, kg | 79.7 (22.3) | 79.0 (21.8) | 80.2 (22.6) | 0.096 |
| GNRI | 99.1 (17.1) | 100.2 (16.8) | 98.3 (17.2) | < 0.001 |
| GCS at admission | 11.2 (4.1) | 12.3 (3.7) | 10.5 (4.3) | < 0.001 |
| SAPS II | 46.0 (14.4) | 41.8 (13.1) | 49.0 (14.5) | < 0.001 |
| WBC, ×10⁹/L | 9.6 (6.9, 13.3) | 9.3 (6.8, 12.8) | 9.8 (7.0, 13.7) | < 0.001 |
| Hemoglobin, g/dL | 9.8 (2.2) | 10.1 (2.2) | 9.6 (2.2) | < 0.001 |
| Platelet, ×10⁹/L | 180.1 (100.5) | 188.7 (102.0) | 174.1 (98.9) | < 0.001 |
| Sodium, mmol/L | 136.6 (5.6) | 136.3 (5.7) | 136.8 (5.6) | 0.014 |
| Calcium, mg/dL | 8.0 (0.9) | 8.1 (0.8) | 7.9 (0.9) | < 0.001 |
| Albumin, g/dL | 3.1 (0.6) | 3.2 (0.6) | 3.0 (0.6) | < 0.001 |
| Comorbidities, n (%) |  |  |  |  |
| Hypertension | 1245 (32.5) | 602 (38.2) | 643 (28.5) | < 0.001 |
| Diabetes | 1366 (35.6) | 528 (33.5) | 838 (37.1) | 0.023 |
| Myocardial infarction | 1010 (26.3) | 437 (27.7) | 573 (25.4) | 0.100 |
| Congestive heart failure | 1553 (40.5) | 658 (41.8) | 895 (39.6) | 0.182 |
| Cerebrovascular disease | 683 (17.8) | 232 (14.7) | 451 (20.0) | < 0.001 |
| Sepsis | 2722 (71.0) | 995 (63.1) | 1727 (76.4) | < 0.001 |
| Liver disease | 789 (20.6) | 235 (14.9) | 554 (24.5) | < 0.001 |
| Malignant cancer | 648 (16.9) | 264 (16.8) | 384 (17.0) | 0.845 |
| Dementia | 234 (6.1) | 68 (4.3) | 166 (7.3) | < 0.001 |
| Outcomes |  |  |  |  |
| Dysphagia, n (%) | N/A | 222 (14.1) | N/A |  |
| ICU length of stay, days | 4.0 (2.1, 7.8) | 3.1 (1.9, 6.0) | 4.7 (2.6, 8.9) | < 0.001 |
| Hospital length of stay, days | 9.8 (5.8, 17.0) | 8.2 (5.1, 14.2) | 10.9 (6.5, 18.9) | < 0.001 |
| ICU mortality, n (%) | 749 (19.5) | 212 (13.5) | 537 (23.8) | < 0.001 |
| Hospital mortality, n (%) | 958 (25.0) | 274 (17.4) | 684 (30.3) | < 0.001 |
| 30-day mortality, n (%) | 1134 (29.6) | 349 (22.1) | 785 (34.7) | < 0.001 |
| 365-day mortality, n (%) | 1765 (46.0) | 613 (38.9) | 1152 (51.0) | < 0.001 |

**Abbreviations:** GNRI, Geriatric Nutritional Risk Index; SAPS II, Simplified Acute Physiology Score II; WBC, white blood cell.
