# Supplementary material for: Association between the geriatric nutritional risk index and dysphagia in older critically ill patients: A retrospective analysis of the MIMIC-IV database
Source: Medicine (Baltimore). 2026 Nov 6;105(45):e51456. doi: 10.1097/MD.0000000000051456 (PMC13747612; doi:10.1097/MD.0000000000051456)
Supplement: Supplementary file 6 [file medi-105-e51456-s006.docx]

**Supplementary Table 6.** Multivariable logistic regression for GNRI and dysphagia in the original dataset (without multiple imputation)

| Variable | n (total) |  | Model 1 | | Model 2 | | Model 3 | | Model 4 | |
| --- | --- | --- | --- | --- | --- | --- | --- | --- | --- | --- |
|  |  | n (%) | OR (95% CI) | P Value | OR (95% CI) | P Value | OR (95% CI) | P Value | OR (95% CI) | *P* Value |
| GNRI | 1527 | 217 (14.2) | 0.97 (0.96–0.98) | <0.001 | 0.97 (0.96–0.98) | <0.001 | 0.97 (0.96–0.98) | <0.001 | 0.97 (0.96–0.98) | <0.001 |
| Quartiles |  |  |  |  |  |  |  |  |  |  |
| Q1 (≤89.88) | 391 | 92 (23.5) | 1(Ref) |  | 1(Ref) |  | 1(Ref) |  | 1(Ref) |  |
| Q2 (89.89-99.19) | 378 | 48 (12.7) | 0.47 (0.32–0.69) | <0.001 | 0.48 (0.33–0.70) | <0.001 | 0.44 (0.29–0.67) | <0.001 | 0.45 (0.30–0.69) | <0.001 |
| Q3 (99.20-109.38) | 378 | 41 (10.8) | 0.40 (0.27–0.59) | <0.001 | 0.40 (0.27–0.59) | <0.001 | 0.40 (0.26–0.63) | <0.001 | 0.42 (0.27–0.66) | <0.001 |
| Q4 (≥109.39) | 380 | 36 (9.5) | 0.34 (0.22–0.52) | <0.001 | 0.34 (0.23–0.52) | <0.001 | 0.33 (0.21–0.53) | <0.001 | 0.34 (0.21–0.55) | <0.001 |
| Trend test | 1527 | 217 (14.2) |  | <0.001 |  | <0.001 |  | <0.001 |  | <0.001 |

Model 1: adjusted for none.

Model 2: adjusted for age, sex, and race.

Model 3: adjusted for model 2 + white blood cell, hemoglobin, platelet, sodium, calcium, hypertension, diabetes, myocardial infarction, congestive heart failure, dementia, cerebrovascular disease, sepsis, liver disease, and malignant cancer.

Model 4: adjusted for model 3 + SAPS II.

**Abbreviations:** GNRI, Geriatric Nutritional Risk Index; OR, odds ratio; CI, confidence interval; SAPS II, Simplified Acute Physiology Score II; Ref, Reference.
