# Supplementary material for: Association between the geriatric nutritional risk index and dysphagia in older critically ill patients: A retrospective analysis of the MIMIC-IV database
Source: Medicine (Baltimore). 2026 Nov 6;105(45):e51456. doi: 10.1097/MD.0000000000051456 (PMC13747612; doi:10.1097/MD.0000000000051456)
Supplement: Supplementary file 7 [file medi-105-e51456-s007.docx]

**Supplementary Table 7.** Multivariable logistic regression for GNRI and dysphagia excluding cerebrovascular disease, cancer, or dementia

| Variable | n (total) |  | Model 1 | | Model 2 | | Model 3 | | Model 4 | |
| --- | --- | --- | --- | --- | --- | --- | --- | --- | --- | --- |
|  |  | n (%) | OR (95% CI) | P Value | OR (95% CI) | P Value | OR (95% CI) | P Value | OR (95% CI) | *P* Value |
| GNRI | 1063 | 119 (11.2) | 0.96 (0.95–0.98) | <0.001 | 0.97 (0.95–0.98) | <0.001 | 0.96 (0.95–0.98) | <0.001 | 0.96 (0.95–0.98) | <0.001 |
| Quartiles |  |  |  |  |  |  |  |  |  |  |
| Q1 (≤89.88) | 240 | 50 (20.8) | 1(Ref) |  | 1(Ref) |  | 1(Ref) |  | 1(Ref) |  |
| Q2 (89.89-99.19) | 255 | 27 (10.6) | 0.45 (0.27–0.75) | 0.002 | 0.46 (0.28–0.77) | 0.003 | 0.44 (0.26–0.76) | 0.003 | 0.45 (0.27–0.78) | 0.004 |
| Q3 (99.20-109.38) | 279 | 19 (6.8) | 0.28 (0.16–0.49) | <0.001 | 0.29 (0.17–0.51) | <0.001 | 0.28 (0.15–0.51) | <0.001 | 0.29 (0.16–0.53) | <0.001 |
| Q4 (≥109.39) | 289 | 23 (8) | 0.33 (0.19–0.56) | <0.001 | 0.33 (0.19–0.57) | <0.001 | 0.3 (0.17–0.55) | <0.001 | 0.31 (0.17–0.56) | <0.001 |
| Trend test | 1063 | 119 (11.2) |  | <0.001 |  | <0.001 |  | <0.001 |  | <0.001 |

Model 1: adjusted for none.

Model 2: adjusted for age, sex, and race.

Model 3: adjusted for model 2 + white blood cell, hemoglobin, platelet, sodium, calcium, hypertension, diabetes, myocardial infarction, congestive heart failure, sepsis, liver disease.

Model 4: adjusted for model 3 + SAPS II.

**Abbreviations:** GNRI, Geriatric Nutritional Risk Index; OR, odds ratio; CI, confidence interval; SAPS II, Simplified Acute Physiology Score II; Ref, Reference.
