# Supplementary material for: Association between the geriatric nutritional risk index and dysphagia in older critically ill patients: A retrospective analysis of the MIMIC-IV database
Source: Medicine (Baltimore). 2026 Nov 6;105(45):e51456. doi: 10.1097/MD.0000000000051456 (PMC13747612; doi:10.1097/MD.0000000000051456)
Supplement: Supplementary file 8 [file medi-105-e51456-s008.docx]

**Supplementary Table 8.** Baseline characteristics before and after propensity score matching by GNRI category in the MIMIC-IV cohort

|  | **Before Matching** | | | | | **After Matching** | | | | |
| --- | --- | --- | --- | --- | --- | --- | --- | --- | --- | --- |
| **Item** | **Total** | **GNRI<91.21** | **GNRI≥91.21** | **P** | **SMD** | **Total** | **GNRI<91.21** | **GNRI≥91.21** | ***P*** | **SMD** |
| n | 1576 | 440 | 1136 |  |  | 730 | 365 | 365 |  |  |
| Dysphagia, n (%) | 222 (14.1) | 102 (23.2) | 120 (10.6) | <0.001 |  | 123 (16.8) | 82 (22.5) | 41 (11.2) | <0.001 |  |
| **Demographics** | | | | | | | | | | |
| Age, years | 77.3 (7.8) | 78.6 (7.9) | 76.8 (7.7) | <0.001 | 0.221 | 78.0 (7.8) | 78.3 (8.0) | 77.8 (7.6) | 0.357 | 0.068 |
| Female, n (%) | 701 (44.5) | 192 (43.6) | 509 (44.8) | 0.675 | 0.024 | 326 (44.7) | 169 (46.3) | 157 (43.0) | 0.372 | 0.066 |
| Race, n (%) | White 1113 (70.6) | White 324 (73.6) | White 789 (69.5) | 0.261 | 0.093 | White 533 (73.0) | White 264 (72.3) | White 269 (73.7) | 0.795 | 0.050 |
| **Comorbidities, n (%)** | | | | | | | | | | |
| Hypertension | 602 (38.2) | 152 (34.5) | 450 (39.6) | 0.063 | 0.105 | 262 (35.9) | 133 (36.4) | 129 (35.3) | 0.758 | 0.023 |
| Diabetes | 528 (33.5) | 102 (23.2) | 426 (37.5) | <0.001 | 0.315 | 194 (26.6) | 98 (26.8) | 96 (26.3) | 0.867 | 0.012 |
| Myocardial infarction | 437 (27.7) | 88 (20.0) | 349 (30.7) | <0.001 | 0.248 | 160 (21.9) | 82 (22.5) | 78 (21.4) | 0.720 | 0.026 |
| Congestive heart failure | 658 (41.8) | 157 (35.7) | 501 (44.1) | 0.002 | 0.173 | 281 (38.5) | 142 (38.9) | 139 (38.1) | 0.819 | 0.017 |
| Cerebrovascular disease | 232 (14.7) | 52 (11.8) | 180 (15.8) | 0.043 | 0.117 | 88 (12.1) | 41 (11.2) | 47 (12.9) | 0.495 | 0.051 |
| Dementia | 68 (4.3) | 26 (5.9) | 42 (3.7) | 0.053 | 0.104 | 40 (5.5) | 21 (5.8) | 19 (5.2) | 0.745 | 0.024 |
| Sepsis | 995 (63.1) | 317 (72.0) | 678 (59.7) | <0.001 | 0.263 | 522 (71.5) | 260 (71.2) | 262 (71.8) | 0.870 | 0.012 |
| Liver disease | 235 (14.9) | 76 (17.3) | 159 (14.0) | 0.101 | 0.090 | 125 (17.1) | 61 (16.7) | 64 (17.5) | 0.768 | 0.022 |
| Malignant cancer | 264 (16.8) | 114 (25.9) | 150 (13.2) | <0.001 | 0.325 | 172 (23.6) | 82 (22.5) | 90 (24.7) | 0.485 | 0.052 |
| **Laboratory tests** | | | | | | | | | | |
| WBC, ×10⁹/L | 10.97 (9.96) | 12.53 (14.99) | 10.37 (7.04) | 0.004 | 0.185 | 11.53 (8.87) | 11.52 (7.84) | 11.54 (9.81) | 0.977 | 0.002 |
| Hemoglobin, g/dL | 10.10 (2.21) | 9.39 (2.03) | 10.37 (2.21) | <0.001 | 0.463 | 9.64 (2.05) | 9.58 (2.00) | 9.70 (2.11) | 0.446 | 0.056 |
| Platelet, ×10⁹/L | 188.75 (102.04) | 188.41 (122.51) | 188.88 (92.96) | 0.942 | 0.004 | 187.23 (111.10) | 186.64 (117.86) | 187.82 (104.07) | 0.885 | 0.011 |
| Sodium, mmol/L | 136.34 (5.67) | 136.33 (6.53) | 136.35 (5.30) | 0.951 | 0.004 | 136.51 (6.06) | 136.49 (6.71) | 136.52 (5.34) | 0.942 | 0.005 |
| Calcium, mg/dL | 8.08 (0.85) | 7.64 (0.91) | 8.26 (0.77) | <0.001 | 0.734 | 7.78 (0.78) | 7.76 (0.80) | 7.79 (0.77) | 0.684 | 0.030 |
| **Scoring systems** | | | | | | | | | | |
| SAPS II | 41.76 (13.08) | 46.70 (14.01) | 39.84 (12.19) | <0.001 | 0.522 | 44.95 (13.00) | 45.21 (12.73) | 44.70 (13.28) | 0.601 | 0.039 |

Note: Data are presented as mean (SD) for continuous variables and number (percentage) for categorical variables. **Abbreviations:** GNRI, Geriatric Nutritional Risk Index; MIMIC-IV, Medical Information Mart for Intensive Care IV; WBC, white blood cell; SAPS II, Simplified Acute Physiology Score II. SMD, standardized mean difference; SMD < 0.10 indicates adequate balance.
