# Supplementary material for: Association between the geriatric nutritional risk index and dysphagia in older critically ill patients: A retrospective analysis of the MIMIC-IV database
Source: Medicine (Baltimore). 2026 Nov 6;105(45):e51456. doi: 10.1097/MD.0000000000051456 (PMC13747612; doi:10.1097/MD.0000000000051456)
Supplement: Supplementary file 9 [file medi-105-e51456-s010.docx]

**Supplementary Table 10.** Multivariable logistic regression for capped GNRI (GNRIc) and dysphagia

| Variable | n (total) |  | Model 1 | | Model 2 | | Model 3 | | Model 4 | |
| --- | --- | --- | --- | --- | --- | --- | --- | --- | --- | --- |
|  |  | n (%) | OR (95% CI) | *P* Value | OR (95% CI) | *P* Value | OR (95% CI) | *P* Value | OR (95% CI) | *P* Value |
| Capped GNRI | 1576 | 222 (14.1) | 0.95 (0.94–0.97) | <0.001 | 0.95 (0.94–0.97) | <0.001 | 0.94 (0.93–0.96) | <0.001 | 0.95 (0.93–0.96) | <0.001 |
| Quartiles |  |  |  |  |  |  |  |  |  |  |
| Q1 (<81.90) | 348 | 88 (25.3) | 1 (Ref) |  | 1 (Ref) |  | 1 (Ref) |  | 1 (Ref) |  |
| Q2 (81.90–89.34) | 413 | 52 (12.6) | 0.43 (0.29–0.62) | <0.001 | 0.42 (0.29–0.62) | <0.001 | 0.40 (0.27–0.61) | <0.001 | 0.42 (0.28–0.63) | <0.001 |
| Q3 (89.35–95.29) | 386 | 39 (10.1) | 0.33 (0.22–0.50) | <0.001 | 0.33 (0.22–0.50) | <0.001 | 0.30 (0.19–0.48) | <0.001 | 0.32 (0.20–0.50) | <0.001 |
| Q4 (≥95.30) | 429 | 43 (10.0) | 0.33 (0.22–0.49) | <0.001 | 0.34 (0.23–0.50) | <0.001 | 0.28 (0.17–0.47) | <0.001 | 0.31 (0.18–0.51) | <0.001 |
| Trend test | 1576 | 222 (14.1) |  | <0.001 |  | <0.001 |  | <0.001 |  | <0.001 |

Model 1: adjusted for none.

Model 2: adjusted for age, sex, and race.

Model 3: adjusted for model 2 + white blood cell, hemoglobin, platelet, sodium, calcium, hypertension, diabetes, myocardial infarction, congestive heart failure, dementia, cerebrovascular disease, sepsis, liver disease, and malignant cancer.

Model 4: adjusted for model 3 + SAPS II.

**Abbreviations:** GNRI, Geriatric Nutritional Risk Index; OR, odds ratio; SAPS II, Simplified Acute Physiology Score II.
