# Supplementary material for: Association between the geriatric nutritional risk index and dysphagia in older critically ill patients: A retrospective analysis of the MIMIC-IV database
Source: Medicine (Baltimore). 2026 Nov 6;105(45):e51456. doi: 10.1097/MD.0000000000051456 (PMC13747612; doi:10.1097/MD.0000000000051456)
Supplement: Supplementary file 10 [file medi-105-e51456-s011.docx]

**Supplementary Table 9.** Weighted baseline characteristics and dysphagia across propensity score weighting methods in MIMIC-IV

|  | **IPTW** | | | **SMRW** | | | **PA** | | | **OW** | | |
| --- | --- | --- | --- | --- | --- | --- | --- | --- | --- | --- | --- | --- |
| **Item** | **GNRI<91.21** | **GNRI≥91.21** | **SMD** | **GNRI<91.21** | **GNRI≥91.21** | **SMD** | **GNRI<91.21** | **GNRI≥91.21** | **SMD** | **GNRI<91.21** | **GNRI≥91.21** | **SMD** |
| Sum of weights | 495.56 | 1138.70 |  | 517.08 | 1136.00 |  | 372.28 | 367.78 |  | 253.81 | 253.81 |  |
| Dysphagia (weighted events), n (%) | 160.7 (32.4) | 123.8 (10.9) |  | 183.5 (35.5) | 120.0 (10.6) |  | 86.0 (23.1) | 38.5 (10.5) |  | 58.0 (22.8) | 27.3 (10.7) |  |
| **Demographics** | | | | | | | | | | | | |
| Age, years | 77.53 (7.37) | 77.42 (7.79) | 0.014 | 77.19 (7.13) | 76.83 (7.67) | 0.049 | 78.43 (7.95) | 78.39 (7.91) | 0.005 | 78.23 (7.96) | 78.23 (7.86) | <0.001 |
| Female, n (%) | 260.3 (52.5) | 511.8 (44.9) | 0.152 | 286.8 (55.5) | 509.0 (44.8) | 0.214 | 169.9 (45.6) | 168.5 (45.8) | 0.004 | 115.9 (45.7) | 115.9 (45.7) | <0.001 |
| Race, n (%) | White 309.8 (62.5) | White 806.1 (70.8) | 0.386 | White 304.3 (58.8) | White 789.0 (69.5) | 0.474 | White 271.0 (72.8) | White 270.0 (73.4) | 0.022 | White 184.4 (72.7) | White 184.4 (72.7) | <0.001 |
| Comorbidities, n (%) | | | | | | | | | | | | |
| Hypertension | 237.2 (47.9) | 440.7 (38.7) | 0.186 | 270.3 (52.3) | 450.0 (39.6) | 0.256 | 135.2 (36.3) | 132.9 (36.1) | 0.003 | 92.9 (36.6) | 92.9 (36.6) | <0.001 |
| Diabetes | 202.5 (40.9) | 380.0 (33.4) | 0.156 | 241.4 (46.7) | 426.0 (37.5) | 0.187 | 94.3 (25.3) | 94.8 (25.8) | 0.010 | 69.2 (27.3) | 69.2 (27.3) | <0.001 |
| Myocardial infarction | 114.4 (23.1) | 318.3 (27.9) | 0.112 | 124.6 (24.1) | 349.0 (30.7) | 0.149 | 80.9 (21.7) | 81.0 (22.0) | 0.007 | 59.1 (23.3) | 59.1 (23.3) | <0.001 |
| Congestive heart failure | 180.3 (36.4) | 483.6 (42.5) | 0.125 | 189.3 (36.6) | 501.0 (44.1) | 0.153 | 144.0 (38.7) | 143.6 (39.0) | 0.007 | 101.2 (39.9) | 101.2 (39.9) | <0.001 |
| Cerebrovascular disease | 49.9 (10.1) | 164.4 (14.4) | 0.133 | 49.1 ( 9.5) | 180.0 (15.8) | 0.192 | 43.7 (11.7) | 40.4 (11.0) | 0.024 | 30.0 (11.8) | 30.0 (11.8) | <0.001 |
| Dementia | 15.9 ( 3.2) | 48.5 ( 4.3) | 0.055 | 12.0 ( 2.3) | 42.0 ( 3.7) | 0.080 | 19.2 ( 5.1) | 17.9 ( 4.9) | 0.013 | 12.3 ( 4.8) | 12.3 ( 4.8) | <0.001 |
| Sepsis | 267.0 (53.9) | 726.2 (63.8) | 0.202 | 247.6 (47.9) | 678.0 (59.7) | 0.238 | 261.3 (70.2) | 256.3 (69.7) | 0.011 | 174.1 (68.6) | 174.1 (68.6) | <0.001 |
| Liver disease | 74.4 (15.0) | 175.8 (15.4) | 0.012 | 73.7 (14.3) | 159.0 (14.0) | 0.007 | 62.9 (16.9) | 62.7 (17.0) | 0.004 | 42.3 (16.7) | 42.3 (16.7) | <0.001 |
| Malignant cancer | 76.4 (15.4) | 200.2 (17.6) | 0.058 | 61.9 (12.0) | 150.0 (13.2) | 0.037 | 84.6 (22.7) | 84.2 (22.9) | 0.004 | 54.6 (21.5) | 54.6 (21.5) | <0.001 |
| Laboratory tests | | | | | | | | | | | | |
| WBC, ×10⁹/L | 10.65 (9.19) | 10.78 (7.91) | 0.015 | 10.03 (6.06) | 10.37 (7.04) | 0.051 | 11.32 (7.69) | 11.57 (9.71) | 0.028 | 11.32 (7.72) | 11.32 (9.10) | <0.001 |
| Hemoglobin, g/dL | 10.07 (2.00) | 10.11 (2.20) | 0.020 | 10.29 (1.94) | 10.37 (2.21) | 0.038 | 9.56 (1.98) | 9.58 (2.05) | 0.008 | 9.69 (2.02) | 9.69 (2.07) | <0.001 |
| Platelet, ×10⁹/L | 191.82 (100.92) | 188.63 (97.26) | 0.032 | 192.95 (92.68) | 188.88 (92.96) | 0.044 | 186.14 (114.31) | 185.55 (103.96) | 0.005 | 186.82 (111.98) | 186.82 (102.23) | <0.001 |
| Sodium, mmol/L | 135.76 (5.82) | 136.26 (5.30) | 0.091 | 135.57 (5.55) | 136.35 (5.30) | 0.144 | 136.35 (6.38) | 136.20 (5.32) | 0.026 | 136.25 (6.37) | 136.25 (5.32) | <0.001 |
| Calcium, mg/dL | 8.67 (1.71) | 8.08 (0.83) | 0.433 | 9.01 (1.78) | 8.26 (0.77) | 0.546 | 7.79 (0.81) | 7.81 (0.77) | 0.017 | 7.87 (0.85) | 7.87 (0.78) | <0.001 |
| Scoring systems | | | | | | | | | | | | |
| SAPS II | 42.90 (12.08) | 42.00 (13.29) | 0.071 | 41.65 (11.09) | 39.84 (12.19) | 0.155 | 44.81 (12.55) | 45.19 (13.45) | 0.030 | 44.41 (12.65) | 44.41 (13.42) | <0.001 |

Note: Values are weighted means (SD) for continuous variables and weighted counts (%) for categorical variables. Dysphagia is reported as weighted events under each weighting method. The “Sum of weights” row reports the sum of the propensity weights. For the IPTW and SMRW methods, covariate balance was incomplete (SMD > 0.10 for several covariates) and extreme weights were present; the corresponding estimates should therefore be interpreted with caution. **Abbreviations:** GNRI, Geriatric Nutritional Risk Index; MIMIC-IV, Medical Information Mart for Intensive Care IV; IPTW, inverse probability of treatment weighting; SMRW, standardized mortality ratio weighting; PA, pairwise algorithm; OW, overlap weighting; WBC, white blood cell; SAPS II, Simplified Acute Physiology Score II; SMD, standardized mean difference.
